# Advancing treatment for perihilar cholangiocarcinoma: role of hepatopancreaticoduodenectomy in small-volume centers

**DOI:** 10.3389/fsurg.2024.1406508

**Published:** 2024-05-14

**Authors:** Sang Yun Shin, Eun Jeong Jang, Sung Hwa Kang, Eun Hwa Park, Kwan Woo Kim

**Affiliations:** Department of Surgery, Dong-A University College of Medicine, Dong-A University Medical Center, Busan, Republic of Korea

**Keywords:** perihilar cholangiocarcinoma, hepatectomy, pancreatoduodenectomy, overall survival rates, recurrence-free survival rates

## Abstract

Hepatopancreaticoduodenectomy (HPD) is a controversial surgical technique for the treatment of perihilar cholangiocarcinoma. This study aimed to clarify the mortality, morbidity, and survival outcomes in patients with perihilar cholangiocarcinoma who underwent HPD at a small-volume hepatobiliary-pancreatic center. This retrospective study included 78 patients with perihilar cholangiocarcinoma who underwent HPD (*n* = 18) or major liver resection with bile duct resection (*n* = 60) at our center between October 2013 and December 2022. The primary endpoints were the in-hospital morbidity and 90-day mortality rates. The secondary endpoints included the recurrence-free and overall survival rates in both groups. Major complications (Clavien–Dindo grade ≥3) were more common in the HPD group (Group 1, 61.1%) than in the major liver resection group (Group 2; 23.3%, *p* = 0.03). The 1-, 3- and 5-year overall survival rates for Groups 1 and 2 were 66.7%, 41.7%, and 27.8% and 79.9%, 44.5%, and 22.7%, respectively (*p* = 0.89). The 1-, 3-, and 5-year recurrence-free survival rates for Groups 1 and 2 were 64.2%, 53.5%, and 35.6% and 85.3%, 46.8%, and 25.0%, respectively (*p* = 0.41). Although morbidity and mortality after HPD are higher than those after other surgeries, our findings suggest that HPD is a feasible treatment option for perihilar cholangiocarcinoma, even in small-volume centers. However, meticulous pre- and perioperative evaluation of the patient's overall health status, quality of life, and prospective advantages are required.

## 1 Introduction

Although several chemotherapy regimens have been developed for various types of bile-duct cancer, surgical resection remains the definitive cure, and surgical techniques vary according to the location of bile-duct cancer. Hepatectomy is usually performed for intrahepatic cholangiocarcinomas, and bile-duct resection (BDR) or pancreaticoduodenectomy (PD) can be performed for extrahepatic cholangiocarcinoma. However, perihilar cholangiocarcinoma, which tends to spread laterally along the hepatoduodenal ligament, cannot be completely removed using hepatectomy or PD alone. Thus, hepatopancreaticoduodenectomy (HPD), in which hepatectomy and PD are performed simultaneously, is appropriate in selected cases. Introduced by Takasaki et al. (1) in 1980, HPD integrates hepatectomy and PD, facilitating the removal of the total extrahepatic biliary system along with the adjacent liver, pancreas, and duodenum. This procedure was a significant leap in the surgical management of gallbladder cancer and has since been applied to perihilar cholangiocarcinoma with modifications to enhance its efficacy and reduce associated risks (1). While HPD offers a potential for cure in selected cases, its complexity leads to considerable morbidity and mortality, making it a controversial option in the surgical oncology (2).

Although the procedure is controversial, and its use is limited to a handful of centers with advanced hepatobiliary-pancreatic surgical skills, perioperative mortality has notably declined, and survival rates have considerably improved over the years (3–5). These studies underline the necessity of high-volume centers where specialized skills can significantly mitigate the inherent risks associated with complex surgeries. Moreover, the application of HPD in small-volume centers has been explored, providing new insights into its feasibility and the meticulous planning required to manage such high-risk procedures effectively. The retrospective nature of studies conducted in these settings highlights the variability in surgical outcomes and emphasizes the importance of patient selection and perioperative management in improving prognosis (6, 7).

This study aimed to provide new insights into the usefulness of this surgical intervention by focusing on the mortality, morbidity, and survival outcomes of patients who underwent HPD for perihilar cholangiocarcinoma in a small-volume hepatobiliary-pancreatic center over approximately a decade. Furthermore, owing to the small number of HPD cases, the outcomes were compared with those of major liver resection for perihilar cholangiocarcinoma performed during the same period to validate the findings.

## 2 Methods

This retrospective study included 78 patients with perihilar cholangiocarcinoma who underwent HPD (*n* = 18) or major liver resection with BDR (*n* = 60) at Dong-A University Medical Center between October 2013 and December 2022.

The definition of HPD varies across studies. In this study, we defined HPD as a surgical technique in which PD was combined with a major hepatectomy involving more than three Couinaud segments. Major hepatectomy for perihilar cholangiocarcinoma involves concomitant resection of the caudate lobe.

Data regarding patients’ demographic characteristics, comorbidities, American Society of Anesthesiologists score, pre- and post-operative laboratory test results, Child–Turcotte–Pugh score, operative findings, post-operative complications, age-adjusted Charlson comorbidity index (A-CCI) (8), and mortality were extracted from the hospital's medical records. The primary endpoints were the in-hospital morbidity and 90-day mortality rates. The secondary endpoints included the recurrence-free and overall survival rates in both groups.

In patients with jaundice or with a preoperative total bilirubin level >3 mg/dl, preoperative biliary decompression (percutaneous transhepatic bile drainage, endoscopic retrograde biliary drainage, or endoscopic nasobiliary drainage) was performed. Portal vein embolization (PVE) using the percutaneous portal venous approach was performed in patients at risk of post-hepatectomy liver failure (PHLF) due to low future remnant liver volume.

Liver failure was classified according to the International Study Group of Liver Surgery criteria (9); “no liver failure” was defined as grade A, whereas PHLF included grades B and C. Post-operative pancreatic fistula (POPF) was defined according to the International Study Group of Pancreatic Fistula criteria (10). Only grades B and C were included in the pancreatic fistula group. Post-operative complications were evaluated using the Clavien–Dindo classification (11), and major morbidity included grade III–V complications.

Resection margins were categorized as R0 (margin-to-tumor distance ≥1 mm), R1 (margin-to-tumor distance <1 mm), or R2 (macroscopically positive margin) (12). The tumor stage was classified according to the American Joint Committee on Cancer TNM 8th edition system (13).

This study was approved by the Institutional Review Board of Dong-A University Medical Center (No. DAUHIRB-23-140, dated August 2, 2023). The requirement for informed consent was waived because of the retrospective nature of the study.

### 2.1 Surgical procedure

A reversed L-shaped incision was made for laparotomy. Individual hilar dissection and extensive lymph node dissection, including lymph node 16, were performed. Additionally, a frozen distal-bile-duct biopsy was performed to determine the need for PD before proceeding with routine PD. During hepatoduodenal ligament dissection, if major vessel invasion was suspected and seemed resectable, concurrent vessel resection and anastomosis were performed. To facilitate liver resection, we used the hanging maneuver of the liver and a Cavitron Ultrasonic Surgical Aspirator (CUSA, Tyco Healthcare, Mansfield, MA, USA). Subsequently, pancreatojejunostomy, hepaticojejunostomy, duodenojejunostomy, and gastrojejunostomy reconstruction were sequentially performed. All surgical procedures were performed by an experienced hepatobiliary surgeon.

### 2.2 Statistics

Data were analyzed using statistical software (IBM SPSS for Windows, Version 26.0; Chicago, IL, USA). Quantitative variables are expressed as mean ± standard deviation and were compared using Student's *t*-test or the Wilcoxon–Mann–Whitney test. Using chi-square or Fisher's exact tests, other quantitative variables were expressed as numbers and percentages. Kaplan–Meier analysis was used to estimate the survival and recurrence rates from the time of surgery. Statistical significance was set at *p* < 0.05.

## 3 Results

### 3.1 Patient characteristics

During the study period, 18 (23.1%) and 60 (76.9%) patients underwent HPD (Group 1) and major liver resection with BDR (Group 2) for perihilar cholangiocarcinoma, respectively.

The preoperative clinical characteristics of all 78 patients are presented in Table 1, and the extent of hepatectomy for perihilar cholangiocarcinoma in each group is presented in Table 2. The mean age in the two groups was 64.2 and 69.2 years, respectively (*p* = 0.02). Biliary decompression and PVE to increase future remnant liver volume were performed in 15 (83.3%) and 45 (75%) and one (5.5%) and two (3.3%) patients in Groups 1 and 2, respectively.

**Table 1:** Preoperative findings.

	Group 1 (*n* = 18)	Group 2 (*n* = 60)	*P*-value
Sex, male/female, *N* (%)	14/4 (77.8/22.2)	47/13 (78.3/21.7)	0.96
Age, mean ± SD	64.2 ± 7.0	69.2 ± 8.2	0.02
BMI, kg/m^2^, mean ± SD	23.1 ± 3.8	23.5 ± 2.7	0.66
A-CCI, median	4.5	5.0	0.04
ASA, *N* (%)			0.49
1	1 (5.6)	1 (1.7)	
2	8 (44.4)	34 (56.7)	
3	9 (50.0)	25 (41.6)	
Biliary decompression, *N* (%)	15 (83.3)	45 (75.0)	0.46
Portal vein embolization, *N* (%)	1 (5.5)	2 (3.3)	0.66
CTP score, median	6.0	6.0	0.82
Laboratory findings, mean ± SD			
Total bilirubin (mg/dl)	1.8 ± 1.4	2.2 ± 1.5	0.33
Albumin (g/dl)	3.7 ± 0.4	3.6 ± 0.5	0.68
CEA (ng/ml)	17.9 ± 60.2	4.9 ± 6.4	0.09
CA 19-9 (U/ml)	481.2 ± 1,347.3	361.7 ± 591.3	0.57

SD, standard deviation; BMI, body mass index; A-CCI, age-adjusted charlson comorbidity index; ASA, American Society of Anesthesiologists; CTP, child–turcotte–pugh; CEA, carcinoembryonic antigen; CA 19-9, carbohydrate antigen 19-9.

**Table 2:** Extent of hepatectomy.

	Group 1 (*n* = 18)	Group 2 (*n* = 60)	*P*-value
Unplanned operation, *N* (%)	12 (66.8)	5 (8.3)	<0.01
Operation time, Mean ± SD	477.4 ± 110.6	378.7 ± 99.0	<0.01
Retrieved numbers of lymph node, Median, range	11.5 (6–26)	8.0 (0–32)	< 0.01
AJCC stage, *N* (%)			0.3
0	1 (5.6)	4 (6.7)	
I	1 (5.6)	15 (25.0)	
II	5 (27.7)	16 (26.7)	
III	11 (61.1)	23 (38.3)	
IV	0 (0)	2 (3.3)	
Resection margin, *N* (%)			0.74
R0	16 (88.8)	48 (80.0)	
R1	1 (5.6)	12 (20.0)	
R2	1 (5.6)	0 (0)	
Intraoperative transfusion, *N* (%)	11 (61.1)	40 (66.6)	0.66
Combined portal vein resection, *N* (%)	2 (11.1)	5 (8.3)	0.71
Combined arterial resection, *N* (%)	1 (5.5)	7 (11.6)	0.45

SD, standard deviation.

In 12 (66.8%) cases, HPD was not planned before the surgery (Table 3), and the surgical strategy was altered based on intraoperative frozen biopsy results. The mean operation time was 477.4 ± 110.6 and 378.7 ± 99.0 min in Groups 1 and 2, respectively (*p* < 0.01), and a median of 11.5 and 8.0 lymph nodes were retrieved in Groups 1 and 2, respectively (*p* < 0.01). The cancer stage was not significantly different between the groups (*p* = 0.30). R0 resection was achieved in 16 (88.9%) and 48 (80%) patients in Groups 1 and 2, respectively (*p* = 0.74). No statistically significant intergroup differences were observed in intraoperative transfusion requirements or concurrent resection of the portal and arterial vessels.

**Table 3:** Perioperative findings.

	Group 1 (*n* = 18)	Group 2 (*n* = 60)	*P*-value
	*N*, (%)	*N*, (%)	0.86
Right trisectionectomy	1 (5.6)	6 (10.0)	
Extended Right hepatectomy	0 (0)	1 (1.7)	
Right hepatectomy	8 (44.4)	21 (35)	
Left trisectionectomy	0 (0)	3 (5.0)	
Extended Left hepatectomy	4 (22.2)	12 (20.0)	
Left hepatectomy	5 (27.8)	17 (28.3)	

SD, standard deviation; AJCC, American Joint Committee on Cancer.

Major complications (Clavien–Dindo Grade ≥3) were more common in Group 1 than in Group 2 (61.1% vs. 23.3%; *p* = 0.03) (Table 4). The most frequent major complication was abnormal fluid collection in the abdominal cavity (e.g., bile, pancreatic juice, or ascites), for which ultrasonography-guided drainage was performed. The average hospital stay was also longer in Group 1 than in Group 2 (25.0 vs. 14.0 days; *p* < 0.01). The incidence of PHLF (grades B and C) was not significantly different between the groups (*p* = 0.83). Grade B and C POPFs occurred in six patients (33.3%) in Group 1. One patient in each group required reoperation. In Group 1, the cause of reoperation was bleeding from the gastroduodenal artery due to pancreatojejunostomy leakage, and in Group 2, the cause of reoperation was wound evisceration.

**Table 4:** Post-operative surgical outcomes.

	Group 1 (*n* = 18)	Group 2 (*n* = 60)	*P*-value
C-D classification ≥3, *N* (%)	11 (61.1)	14 (23.3)	0.03
Hospital stays, Median (range)	25 (11–91)	14 (6.81)	<0.01
PHLF ≥ grade B, *N* (%)	4 (22.2)	12 (20.0)	<0.01
POPF ≥ grade B, *N* (%)	6 (33.3)	N/A	N/A
In-hospital reoperation, *N* (%)	1 (5.5)	1 (1.6)	0.36
30-day mortality, *N* (%)	2 (11.1)	2 (3.3)	0.18
90-day mortality, *N* (%)	3 (16.6)	3 (5.0)	0.10

C-D, Clavien–Dindo; SD, standard deviation; PHLF, post hepatectomy liver failure; POPF, postoperative pancreatic fistula; N-A, not applicable.

The 30- and 90-day mortality rates in Groups 1 and 2 were 11.1% (*n* = 2) and 3.3% (*n* = 2) and 16.6% (*n* = 3) and 5% (*n* = 3), respectively, and the differences were not statistically significant. In Group 1, the causes of 90-day mortality were post-operative acute respiratory distress syndrome (*n* = 1), pancreatojejunostomy leakage (*n* = 1), and liver failure (*n* = 1). In Group 2, the causes of 90-day mortality were liver failure (*n* = 2) and multi-organ failure (*n* = 1).

### 3.2 Survival outcomes

**Figure 1:**
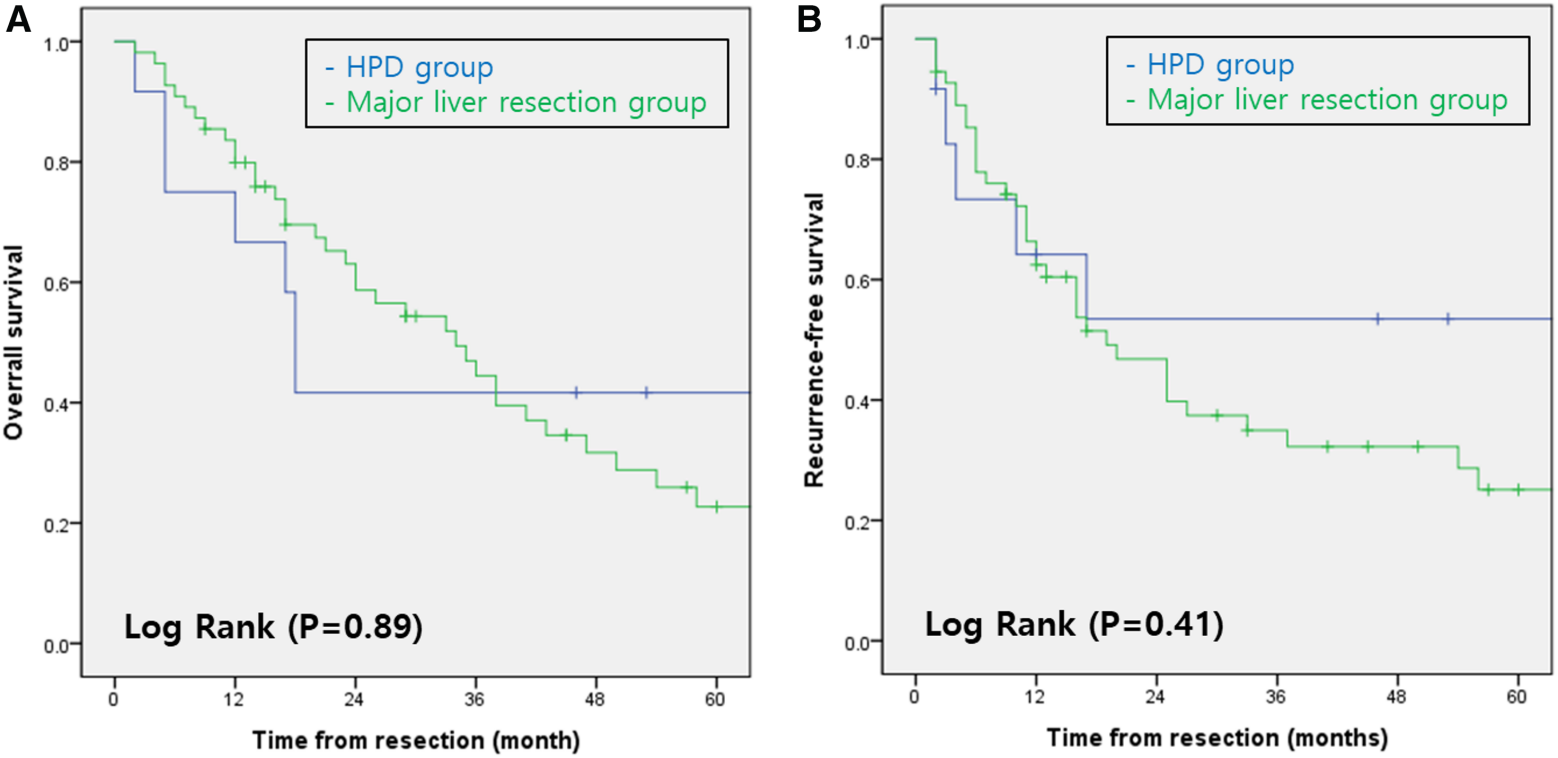
(**A**) overall survival rates of patients with perihilar cholangiocarcinoma according to type of surgery. (**B**) Recurrence-free survival rates of patients with perihilar cholangiocarcinoma according to type of surgery. HPD, Hepatopancreaticoduodenectomy.

The 1-, 3-, and 5-year overall survival rates in Groups 1 and 2 were 66.7%, 41.7%, and 27.8% and 79.9%, 44.5%, and 22.7%, respectively. The overall survival rates did not differ significantly between the two groups (*p* = 0.89; Figure 1A). The 1-, 3-, and 5-year recurrence-free survival rates in Groups 1 and 2 were 64.2%, 53.5%, and 35.6% and 85.3%, 46.8%, and 25.0%, respectively. The recurrence-free survival rates did not differ significantly between the two groups (*p* = 0.41; Figure 1B).

**Figure 2:**
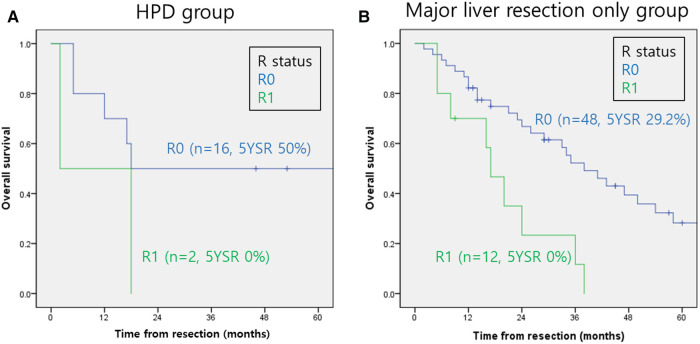
Overall survival rates of perihilar cholangiocarcinoma according to the R status. (**A**) HPD group. (**B**) Major liver resection only group. HPD, Hepatopancreaticoduodenectomy.

The overall survival rates for perihilar cholangiocarcinoma according to R status in both groups are shown in Figure 2. The 5-year overall survival rates according to the R status in Group 1 were 50% and 0% for R0 and R1, respectively (Figure 2A). The 5-year overall survival rates according to the R status in Group 2 were 29.2% and 0% for R0 and R1, respectively (Figure 2B). Patient outcomes after R1 resection were extremely poor in both groups (*p* < 0.01).

## 4 Discussion

In this study, comparing groups 1 and 2 validated the surgical and oncological outcomes of HPD despite the small number of cases of perihilar cholangiocarcinoma in Group 1. Theoretically, HPD, which merges major hepatectomy with PD, is the sole curative option for bile-duct carcinoma that has extensively spread horizontally, afflicting both the hepatic hilum and the intrapancreatic bile duct (6).

In this study, HPD was not anticipated preoperatively in 12 patients, which represents 66.8% of cases. This rate exceeds what has been reported in the literature, where unplanned HPD occurs in 25%–46% of cases as shown by D'Souza et al. (7) and Aoki et al. (14). The preoperative anticipation of HPD presents significant difficulties owing to the nature of tumor proliferation, which tends to follow a vertical path along the mucosal lining of the bile duct (2). Despite advancements in preoperative imaging studies, these details often remain hidden and are typically revealed intraoperatively through a frozen-section biopsy. These considerations explain why there were many unplanned surgeries and only one case of preoperative PVE in this study.

Although the occurrence of unplanned HPD in our study was higher than in prior reports, the associated complications such as PHLF (22.2%), POPF (33.3%), in-hospital morbidity (5.5%) and 90-day mortality (16.6%) aligned with previous studies (6, 7, 15). PHLF is an important predictor of mortality after perihilar cholangiocarcinoma resection. we observed PHLF in 22.2% of patients in Group 1% and 20% in Group 2, which sits within the reported variability of 4%–66% seen in similar procedures. Ebata et al. (5) and Aoki et al. (14) have similarly reported variable rates, which suggests that differing definitions of PHLF could impact the reported incidence across studies.

Furthermore, we encountered a 33.3% rate of POPF in our HPD procedures, reflecting the complex nature of the surgery. This is somewhat higher than the approximately 20% incidence for clinically significant POPF (grades B and C) following PD, as delineated by the criteria from the International Study Group of Pancreatic Fistula (16, 17).

The risk of POPF is higher in patients with soft pancreatic texture and narrow pancreatic ducts, particularly among patients who undergo HPD. POPF is associated with increased morbidity and mortality after PD. Unfortunately, we encountered one case each of mortality due to PHLF and POPF in Group 1.

Patients in Group 1 were younger and had a lower mean A-CCI than did those in Group 2. The differences in age (*p* = 0.02) and A-CCI (*p* = 0.04) could be attributed to the surgeon’s selection of healthier patients to improve the surgical outcomes of HPD, which is complex and is associated with higher morbidity and mortality. Therefore, owing to the frequency of unplanned HPD, age and A-CCI are essential factors that must be considered when deciding to proceed with HPD. The longer operation time and larger lymph node retrieval numbers in Group 1 were expected and are indicative of the complexity of the HPD. Furthermore, the higher incidence of major complications and longer mean duration of hospital stay in Group 1 (Table 4) were expected, demonstrating the increased demand for post-operative care and the potential need for more comprehensive management strategies for patients undergoing HPD.

Despite the small cohort in Group 1, the lack of significant intergroup differences in the overall and recurrence-free survival rates suggests that this surgery has a place in the treatment protocol, despite the complexity of HPD (Figure 1). The overall and recurrence-free survival rates for perihilar cholangiocarcinoma, regardless of the surgery type, are consistent with those in several other studies (14, 18–20). This validates the results of this study despite its small sample size.

Because the risk factors affecting the prognosis of perihilar cholangiocarcinoma, such as tumor invasion depth (21), lymph-node metastasis (22), and the degree of histologic differentiation and recurrence (19), are well established, they were not specifically investigated in this study. However, a clear resection margin (R0) is the most important prognostic factor affecting long-term survival rates (15, 19). In this study, the dismal prognosis following R1 resection across groups reinforced the critical need to achieve negative margins (Figure 2). Despite the small number of cases, these results suggest that HPD is a justifiable treatment for perihilar cholangiocarcinoma, as the survival outcomes did not differ significantly from those of major liver resection with BDR. This indicates that the additional risks and complexities of HPD are counterbalanced by its potential to improve survival outcomes.

The higher complication rates and longer duration of hospital stay in Group 1 highlight the need for careful patient selection and perhaps more aggressive perioperative management. When discussing treatment options and the potential risks and benefits of HPD, these findings should be communicated to patients and their families.

Owing to the retrospective nature of this study and the small-volume center data, further studies with larger sample sizes are required to confirm the findings of this study.

## 5 Conclusions

Although the morbidity and mortality after HPD are higher than those after other surgeries, our findings suggest that HPD is a feasible treatment option for perihilar cholangiocarcinoma, even in small-volume centers. However, meticulous pre- or perioperative evaluations of the patient's overall health status and prospective advantages are required.

## Data Availability

The original contributions presented in the study are included in the article/Supplementary Material, further inquiries can be directed to the corresponding author.
